# Bio-Inspired Observability Enhancement Method for UAV Target Localization and Sensor Bias Estimation with Bearing-Only Measurement

**DOI:** 10.3390/biomimetics10050336

**Published:** 2025-05-20

**Authors:** Qianshuai Wang, Zeyuan Li, Jicheng Peng, Kelin Lu

**Affiliations:** School of Automation, Southeast University, Nanjing 210096, China; xiaoshuaiwang703@gmail.com (Q.W.); 220242202@seu.edu.cn (Z.L.); pengjicheng@seu.edu.cn (J.P.)

**Keywords:** bio-inspiration, observability enhancement, trajectory optimization, sensor bias, target localization

## Abstract

This paper addresses the problem of observability analysis and enhancement for UAV target localization and sensor bias estimation with bearing-only measurement. Inspired by the compound eye vision, a bio-inspired observability analysis method is proposed for stochastic systems. Furthermore, a performance metric that can be utilized in UAV trajectory optimization for observability enhancement of the target localization system is formulated based on maximum mean discrepancy. The performance metric and the distance of the UAV relative to the target are utilized as objective functions for trajectory optimization. To determine the decision variables (the UAV’s velocity and turn rate) for UAV maneuver decision making, a multi-objective optimization framework is constructed, and is subsequently solved via the nonlinear constrained multi-objective whale optimization algorithm. Finally, the analytical results are validated through numerical simulations and comparative analyses. The proposed method demonstrates superior convergence in both target localization and sensor bias estimation. The nonlinear constrained multi-objective whale optimization algorithm achieves minimal values for both generational distance and inverted generational distance, demonstrating superior convergence and diversity characteristics.

## 1. Introduction

In recent years, unmanned aerial vehicles (UAVs) have gained significant popularity owing to their portability [1], cost-effectiveness [2], and maneuverability [3]. These characteristics render UAVs particularly suitable for specialized missions including reconnaissance [4], search and rescue operations [5,6], aerial mapping [7], and military precision strikes [8]. Target localization [9], key to these tasks, primarily involves estimating target positions through onboard sensors. However, UAV operational capabilities are constrained by limited payload capacity and endurance, restricting the deployment of heavy or high-power sensors [10]. Consequently, lightweight sensors have become the predominant solution for such scenarios.

While lightweight sensors demonstrate distinct advantages in specific scenarios, they are typically limited to measuring the bearing or line-of-sight angle. These measurements are frequently compromised by sensor bias [11] and measurement noise [12], constituting the typical bearing-only target localization problem [13]. Doğançay and Kutluyıl [14] proposed a total least-squares algorithm for the bearing-only target localization problem. Frew and Eric [15] established the necessary conditions for bearing-only target localization, providing a rigorous theoretical foundation. The target position is estimated via a filtering approach that incorporates bearing information. The Kalman filter, in particular, offers significant advantages in handling white noise [16]. In [17], a novel ensemble Kalman filter was proposed for target localization with bearing-only measurement.

Given that the relative position relationship between the UAV and the target directly influences the performance of state estimation [18], optimizing the UAV trajectory is essential to improve the accuracy of target localization. Nardone, Aidala [19], and Hammel [20] derived the observability conditions for two-dimensional target localization, demonstrating that UAV acceleration must satisfy particular requirements to guarantee sufficient observability. This seminal work established the theoretical foundation for subsequent advancements in bearing-only target localization research. To enhance two-spacecraft observability, Zhou [21] proposed adding a third spacecraft, with an extended Kalman filter validating the improved observability convergence relationship through numerical simulations. In [22], a reinforcement learning-based guidance system was developed for angles-only rendezvous and proximity operation missions while guaranteeing observability and safety through optimized trajectory planning and robust constraint satisfaction. An expanding corpus of scholarly work has emerged, focusing on enhancing state estimation accuracy through improved observability analysis techniques. Anjaly and Ratnoo [23] derived an observability metric based on the Fisher information matrix and the Cramér–Rao lower bound to quantitatively assess UAV maneuver effectiveness. A notable advantage is the practical application to UAV rendezvous scenarios, where a cooperative leader maneuver is designed to maximize observability, supported by error ellipsoid plots that validate the analytical findings. Fujiwara [24] extended this approach by integrating a quantitative observability metric derived from the Fisher information matrix, which was optimized as a cost function to enhance system observability. Its key innovative contributions include integrating the Fisher information matrix into the cost function to quantify observability and deriving semi-analytic gradients and Hessians for efficient convergence under impulsive maneuvers. Yang [25] formulated a constrained framework for UAV trajectory optimization that maximizes the determinant of the observability Gramian matrix to enhance system observability. The main advantages over conventional calibration techniques are the systematic optimization of observability and real-time applicability, supported by numerical and experimental validation. He [26] developed a geometric approach for UAV trajectory optimization with bearing-only measurements, employing the relative distance and separation angle as the cost function.

Existing approaches frequently disregard system process noise and assume ideal control conditions where a UAV can perfectly execute optimized trajectories through high-performance controllers. In practice, target observability is fundamentally governed by the UAV–target relative geometry [27,28], a relationship frequently compromised by process noise. Consequently, an observability analysis method that incorporates process noise is essential for UAV trajectory optimization. Ugrinovskii [29] proposed an observability metric for stochastic systems based on the relative entropy functional. This metric quantifies the difference between two probability distributions and provides a theoretical foundation for evaluating system observability under uncertainty. In [30], a method based on generalized polynomial chaos was employed for the observability analysis of stochastic systems. In [31], a method based on the empirical observability Gramian was proposed for the observability analysis of stochastic nonlinear systems. The aforementioned methods characterize the impact of process noise on observability via the observability matrix. However, the approaches remain constrained by the observability matrix, resulting in diminished robustness against process noise disturbances. Consequently, it is unsuitable for UAV trajectory optimization in bearing-only target localization scenarios.

Additionally, trajectory optimization frequently requires a trade-off between observability and other performance metrics. These multi-objective optimization problems can be effectively addressed through metaheuristic algorithms (MHAs) [32,33,34]. Nature-inspired MHAs address optimization problems by emulating biological behaviors or physical phenomena [35]. These algorithms typically initialize with randomly generated populations, which then undergo iterative evolutionary processes. Through this approach, the population gradually converges [36], ultimately yielding an approximate global optimum solution [37]. Karimi and Pourtakdoust [38] proposed a dynamic hybrid particle swarm optimization algorithm for real-time motion planning of UAVs in complex terrains with stochastic threats. Akopov [39] developed a parallel biobjective real-coded genetic algorithm to address maneuverability challenges in multiagent fuzzy transportation systems with conflicting objectives: minimizing traffic accidents and maximizing traffic flow. Coleman [40] developed a UAV trajectory optimization method employing control barrier functions to enhance target observability, where unobservable regions are modeled as constraints.

Inspired by the compound eye vision [41,42], this study presents a bio-inspired trajectory optimization method, that incorporates process noise, for bearing-only target localization. The contributions of this work are summarized as follows:

(1) Inspired by [26,43], we first derive the observability condition for a deterministic system through the geometric observability analysis method. The concept is subsequently extended to stochastic systems to establish the distributional observability condition. Finally, leveraging a data-driven approach, distributional observability is quantitatively analyzed through maximum mean discrepancy (MME). Based on the quantitative metrics of distributional observability, an optimization model for bearing-only target localization is proposed. The superiority of the proposed method is demonstrated through a comprehensive comparison with a traditional optimization model. The results demonstrate the performance and effectiveness of our approach in addressing the bearing-only target localization problem.
(2) We transform trajectory optimization into a multi-objective nonlinear programming problem, where the quantitative metric of distributional observability and the distance of the UAV relative to the target are utilized as the set of objective functions. The optimal set of decision variables (the UAV’s speed and turn rate), that satisfy the platform’s performance constraints, is determined by minimizing the defined objective functions. Drawing on the methodology proposed in [44], a control barrier function is constructed as a nonlinear constraint to ensure the UAV remains outside unobservable regions.
(3) A nonlinear constrained multi-objective whale optimization algorithm (NCMOWOA) is proposed to address the multi-objective nonlinear programming problem. This algorithm improves the multi-objective whale optimization algorithm [45] by incorporating a nonlinear constraint into the optimization model. Specifically, the nonlinear constrained elitist selection strategy (NCESS) is utilized to select solutions at each iteration, ensuring that the solutions satisfy the nonlinear constraint. In this study, the NCMOWOA is compared with several metaheuristic algorithms, including the multi-objective particle swarm optimization algorithm (MOPSOA) [46], nondominated sorting genetic algorithm II (NSGA-II) [47], multi-objective exponential distribution optimization algorithm (MOEDOA) [48], and nondominated sorting genetic algorithm III (NSGA-III) [49]. The comparative analysis demonstrates the performance of NCMOWOA in addressing bearing-only target localization problems.

The remainder of the paper is organized as follows: Section 2 presents the kinematics and the measurement model for the UAV. Section 3 improves the bio-inspired distributional observability analysis method and proposes a performance metric. In Section 4, the UAV trajectory optimization problem is formulated as a multi-objective nonlinear programming problem and subsequently resolved by the NCMOWOA. Finally, the numerical simulations and conclusions presented herein demonstrate that: (1) the proposed method exhibits superior convergence in both target localization and sensor bias estimation tasks, and (2) the NCMOWOA achieves optimal performance with minimal generational distance (GD) and inverted generational distance (IGD) values, confirming its outstanding convergence and diversity characteristics.

## 2. System Modeling

**Figure 1:**
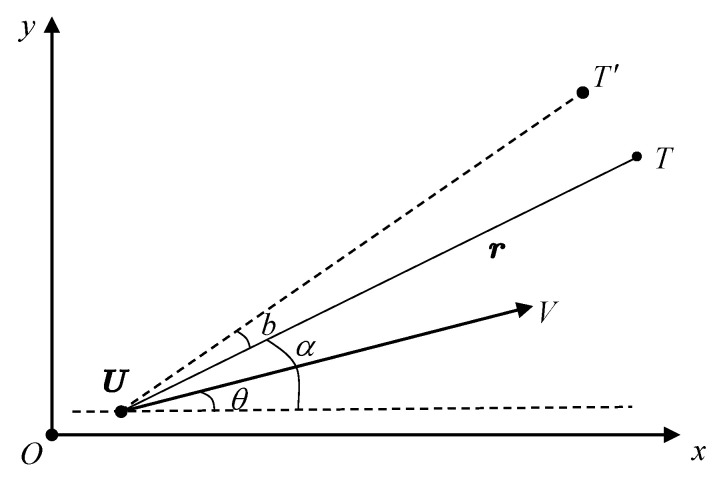
Geometric relationship between the UAV and target in a 2-D inertial coordinate system.

In this paper, we study the problem of target localization via a UAV platform at a fixed altitude with bearing-only measurements within the 2-D coordinate system. As illustrated in Figure 1, U denotes the position of the UAV, *V* represents the velocity of the UAV, *b* signifies the constant bias in the bearing measurement, θ is the heading angle of the UAV, α indicates the bearing angle of the target relative to the UAV, *T* denotes the true target, $T^{\prime }$ represents the false target detected by the UAV due to the constant bias in the bearing measurement, and $r={\left[r_{1},r_{2}\right]}^{T}$ signifies the relative position vector between the UAV and the target.

### 2.1. UAV Kinematics

The UAV within a 2-D inertial coordinate system is given by:

(1) $$\dot{p}=\left[\begin{array}{c} {\dot{p}}_{1}\\ {\dot{p}}_{2} \end{array}\right]=\left[\begin{array}{c} V\cos(\theta )\\ V\sin(\theta ) \end{array}\right]+\left[\begin{array}{c} \epsilon_{1}\\ \epsilon_{2} \end{array}\right]$$

The low-level flight control system manages the trajectory of the UAV by controlling its velocity and turn rate. The input *u* can be expressed as

(2) $$u=\left[\begin{array}{c} u_{1}\\ u_{2} \end{array}\right]=\left[\begin{array}{c} V\\ \dot{\theta } \end{array}\right]$$

where $p={\left[p_{1},p_{2}\right]}^{T}$ represents the position of the UAV in the inertial reference frame, $V\in [V_{\min},V_{\max}]$ denotes the velocity of the UAV, $V_{\min}$ and $V_{\max}$ represent the minimum and maximum velocity, respectively, constrained by the engine performance of the UAV during low-altitude flight, θ denotes the heading angle of the UAV, and $\epsilon ={\left[\epsilon_{1},\epsilon_{2}\right]}^{T}$ is the process noise, assumed to follow Gaussian distribution with zero mean and covariance matrix *Q*. In practice, the turn rate is constrained by physical limitations as

(3) $$∥\theta_{k+1}-\theta_{k}∥\leq \Delta \theta \overset{\Delta }{\;=}\omega_{\max}\Delta t$$

where $\omega_{\max}$ is the maximum turn rate of UAV, and Δt represents the sampling time.

**Remark 1.**  *The UAV is assumed to incorporate an advanced autopilot system with velocity and altitude tracking capabilities. The objective is to design control inputs and target localization algorithms within this control framework, where the inputs are restricted to 2-D maneuvers. Additionally, the UAV’s flight trajectory is optimized through these control inputs to improve the accuracy of target localization.*

### 2.2. Measurement Model

At time step *k*, the system measurement model can be expressed as

(4) $$\begin{array}{c} Z_{k}=\arctan\left(\frac{r_{2,k}}{r_{1,k}}\right)+b+\upsilon_{k}\\ r_{1,k}=x_{T,1,k}-p_{1}\\ r_{2,k}=x_{T,2,k}-p_{2} \end{array}$$

where $x_{T,k}={\left[x_{T,1,k},x_{T,2,k}\right]}^{T}$ denotes the target position at time step *k*, $r_{k}={\left[r_{1,k},r_{2,k}\right]}^{T}$ represents the relative position vector at time step *k*, $\upsilon_{k}$ is the measurement noise at time step *k*, which follows the Gaussian distribution with zero mean and variance *R*, and $Z_{k}$ corresponds to the measured line-of-sight angle at time step *k*.

According to (4), the relative position $r_{k}$ between the UAV and the target cannot be directly obtained from the measurement. The UAV must perform a series of maneuvers to ensure a sufficient degree of observability. To improve target localization accuracy, the constant bias *b* and the target position $x_{T,k}$ are jointly estimated. Various observability analysis methods (such as the observability matrix [50], observability Gramian [51], Lie derivative method [52], and Fisher information matrix [53]) are available, as detailed below.

The observability of a linear system can be assessed using the observability matrix method, formulated as

(5) $$O={\left[C,CA,CA^{2},\;\ldots \;,CA^{n-1}\right]}^{T}$$

where *n* is the dimension of state vector, *C* denotes the output matrix, *A* represents the system matrix, and *O* is the observability matrix. The system is observable if and only if rank(O)=n.

The observability of a linear system can alternatively be analyzed using the observability Gramian matrix method. The observability matrix *O* defined in (5) can be equivalently expressed through the observability Gramian $W_{o}$

(6) $$W_{o}\left(T\right)=\int_{0}^{T}e^{A^{T}t}C^{T}Ce^{At}dt$$

where $W_{o}\left(T\right)\in R^{n\times n}$ is the observability Gramian over time horizon *T*. The system is completely observable if and only if $W_{o}\left(T\right)$ is positive definite for some T>0.

For nonlinear systems, observability can be analyzed using the Lie derivation. Consider a nonlinear system in the form

(7) $$\left\{\begin{array}{c} \dot{x}=f\left(x\right)\\ y=h(x) \end{array}\right.$$

where $x\in R^{n}$ is the state vector, $y\in R^{p}$ is the output, *f* is the vector field, and *h* is the output function. The observability analysis involves constructing the observability Lie derivatives, formulated as

(8) $$L_{f}^{0}h\left(x\right)=h\left(x\right)$$

(9) $$L_{f}^{k}h\left(x\right)=\frac{\partial L_{f}^{k-1}h\left(x\right)}{\partial x}f\left(x\right),\;k\geq 1$$

The observability matrix O(x) is then given by:

(10) $$O\left(x\right)=\left[\begin{array}{c} dL_{f}^{0}h\left(x\right)\\ dL_{f}^{1}h\left(x\right)\\ \vdots \\ dL_{f}^{n-1}h\left(x\right) \end{array}\right]$$

where *d* denotes the differential operator. The system is locally observable if rank(O(x))=n in a neighborhood of x.

For stochastic systems, observability can be analyzed through the Fisher information matrix approach. Consider the stochastic nonlinear system

(11) $$\left\{\begin{array}{c} dx_{t}=f\left(x_{t}\right)dt+G\left(x_{t}\right)dw_{t}\\ y_{t}=h\left(x_{t}\right)+v_{t} \end{array}\right.$$

where $x_{t}\in R^{n}$ is the state vector, $y_{t}\in R^{p}$ is the measurement vector, $w_{t}$ and $v_{t}$ are process and measurement noise (Wiener processes), and $G(x_{t})$ is the noise gain matrix.

The Fisher information matrix $I(x_{t})$ for observability analysis is given by

(12) $$I\left(x_{t}\right)=E\left[\left(\frac{\partial \log p(y_{0:T}|x_{0})}{\partial x_{0}}\right){\left(\frac{\partial \log p(y_{0:T}|x_{0})}{\partial x_{0}}\right)}^{T}\right]$$

where $p(y_{0:T}|x_{0})$ is the conditional probability density of measurements $y_{0:T}$ given initial state $x_{0}$. The system is stochastically observable if $I(x_{t})$ is positive definite.

**Remark 2.**  *The relative position $r_{k}$ obtained by the UAV through the onboard camera is defined in the inertial coordinate frame with the UAV itself as the origin, rather than the target’s position in the global inertial coordinate frame. This paper assumes that the UAV carries a high-precision inertial measurement unit with an optimized attitude estimation filter that provides precision attitude data. The inertial measurement unit output inevitably contains noise, which is incorporated into ϵ.*

## 3. Bio-Inspired Distributional Observability Analysis

This section begins by analyzing the observability condition of a deterministic system. Subsequently, deterministic observability is extended to distributional observability, and the quantitative metric of distributional observability is established.

### 3.1. Observability Analysis for Deterministic System

Disregarding process noise, the discrete-time deterministic UAV kinematics and output are constructed as follows

(13) $$\begin{array}{c} p_{k+1}=f\left(p_{k},v_{k}\right)\\ z_{k}=h\left(p_{k}\right) \end{array}$$

where $p_{k}\in X\subset R^{2}$ is the UAV position at time step *k*, f:X→X represents UAV kinematics, $z_{k}\in Y\subset R$ indicates the output, which is the bearing angle of the UAV relative to the target at time step *k*, and h:X→Y denotes the output map.

**Figure 2:**
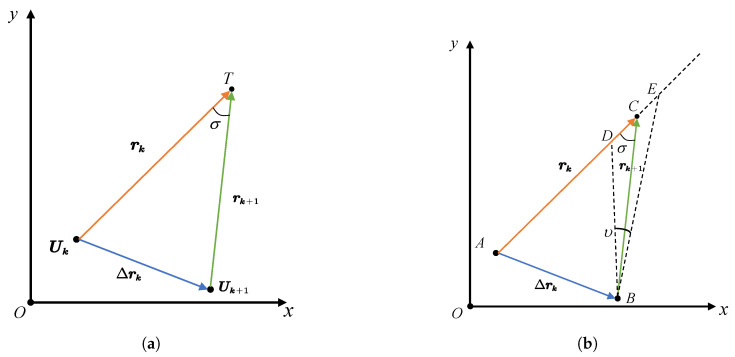
Geometric relationship between the UAV and target at time steps *k* and k+1 within an inertial coordinate system. (**a**) Measurement noise is ignored. (**b**) Measurement noise is taken into account.

The UAV performs the maneuver at time step *k* under the control input. The maneuver $v_{k}$ can be expressed as

(14) $$\begin{array}{c} v_{k}=\left(V_{k}\cos\left(\theta_{k}\right)\Delta t,V_{k}\sin\left(\theta_{k}\right)\Delta t\right)\\ \theta_{k}=\theta_{k-1}+u_{2,k}\Delta t\\ V_{k}=u_{1,k} \end{array}$$

where Δt is the sample time and $u_{k}={\left[u_{1,k},u_{2,k}\right]}^{T}$ denotes the control input at time step *k*. As shown in Figure 2a, the UAV moves from position $U_{k}$ to position $U_{k+1}$ under the maneuver $v_{k}$, where σ denotes the separation angle and $\Delta r_{k}$ denotes the displacement vector.

When σ>0 and given a fixed value of $r_{k}$, the target position can be accurately determined by triangulating the relative position vector $r_{k+1}$. When σ=0, the measurements at time steps *k* and k+1 are nearly identical. In this case, only when the UAV maneuvers between two consecutive time steps such that the separation angle σ>0 can effective target localization be achieved.

Considering the influence of measurement noise, the geometric relationship between the UAV and the target is illustrated in Figure 2b, and the target localization error is denoted as δρ. The trajectory optimization problem can be reformulated as an optimal control problem, where the goal is to find the optimal controller inputs at each time step to minimize the target localization error δρ. The objective function for optimization is expressed as follows

(15) $$J=\frac{{∥r_{k+1}∥}^{2}}{\sin^{2}\sigma }$$

**Remark 3.**  *Analysis of the geometric relationship between the UAV and the target reveals that maximizing $\sin^{2}\sigma$ and minimizing $∥r_{k+1}{∥}^{2}$ are a pair of conflicting objectives. Owing to the relatively large distance between the UAV and the target, the change in $\sin^{2}\sigma$ between two consecutive time steps is not significant, and the value of $∥r_{k+1}{∥}^{2}$ remains approximately constant. Therefore, (15) can be simplified as*

(16) $$J=\frac{1}{\sigma^{2}}$$

Based on (16), the observability condition for the system over two consecutive time steps is derived, requiring the UAV to perform a maneuver $v_{k}$ such that the separation angle is greater than zero, that is

(17) $${∥z_{k+1}-z_{k}∥}_{2}>0$$

where ${∥\cdot ∥}_{2}$ represents the Euclidean norm, retaining the same meaning throughout the context below.

To provide a clearer description of distributional observability, we sample the system T∈N times at positions $p_{k}$ and $p_{k+1}$, obtaining the output trajectories of the system at these two positions as

(18) $$\begin{array}{c} \gamma \left(p_{k},T\right)=\left(h\left(\phi \left(p_{k},1\right)\right),\;\ldots \;,h\left(\phi \left(p_{k},T\right)\right)\right)\in Y^{T}\\ \gamma \left(p_{k+1},T\right)=\left(h\left(\phi \left(p_{k+1},1\right)\right),\;\ldots \;,h\left(\phi \left(p_{k+1},T\right)\right)\right)\in Y^{T} \end{array}$$

where $\phi (p_{k},t)$ represents the result of the *t*-th sampling at position $p_{k}$. Since process noise is not considered, the sampling results at the same position remain identical regardless of the number of samples, i.e., all the results are equal. Equation (18) is equivalent to

(19) $$\gamma \left(p_{k},T\right)\neq \gamma \left(p_{k+1},T\right)⟺h\left(p_{k}\right)\neq h\left(p_{k+1}\right)$$

Importantly, according to (19), the deterministic observability condition can be obtained by output trajectories as follows

**Definition 1.**  *At time step k, the UAV transitions from $p_{k}$ to $p_{k+1}$ under the maneuver $v_{k}$. We state that the target is observable, and $v_{k}$ is considered an effective maneuver if $h\left(p_{k}\right)\neq h\left(p_{k+1}\right)$.*

The effective maneuver is defined as a controlled motion that improves target observability. The distributional observability condition for a stochastic system will be presented later.

### 3.2. Bio-Inspired Distributional Observability Analysis for Stochastic System

In stochastic systems, observability follows a certain distribution. Distributional observability, as the expanded notion of observability, can be effectively computed in a data-driven manner. First, the discrete-time stochastic UAV kinematics and output are constructed as follows

(20) $$\begin{array}{c} P_{k+1}=F\left(P_{k},V_{k}\right)\\ Z_{k}=H\left(P_{k},\upsilon_{k}\right)\\ \begin{array}{c} V_{k}={\left[\left(\epsilon_{1,k}+V_{k}\cos\left(\theta_{k}\right)\right)\Delta t,\left(\epsilon_{2,k}+V_{k}\sin\left(\theta_{k}\right)\right)\Delta t\right]}^{T} \end{array} \end{array}$$

where $P_{k}$ represents the position of the UAV at time step *k* and $V_{k}$ is the distributional maneuver.

**Figure 3:**
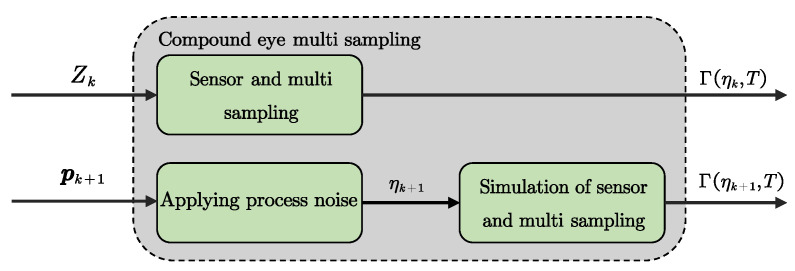
Flowchart of the bio-inspired data acquisition approach.

The UAV cannot execute deterministic maneuvers due to the process noise, which implies the existence of distributional positions $\eta_{k},\eta_{k+1}$ defined on X, $P_{k}∽\eta_{k},P_{k+1}∽\eta_{k+1}$. Stochastic behaviors cannot be adequately characterized by individual measurements. Drawing inspiration from the compound eye vision in insects. A novel data acquisition approach inspired by the compound eye is proposed, which is illustrated in Figure 3.

As shown in Figure 3, $p_{k+1}$ denotes the position derived from the control input $u_{k}$. We perform multiple samples at position $P_{k}$, with $\Phi (\eta_{k},t)$ denoting the result of the *t*-th sampling at the distributional position $\eta_{k}$. Then, the output trajectories of the system at time steps k,k+1 can be expressed as follows

(21) $$\begin{array}{c} \Gamma \left(\eta_{k},T\right)=\left(H\left(\Phi \left(\eta_{k},T\right),\upsilon_{k}\right),\;\ldots \;,H\left(\Phi \left(\eta_{k},T\right),\upsilon_{k}\right)\right)\in Y^{T}\\ \Gamma \left(\eta_{k+1},T\right)=\left(H\left(\Phi \left(\eta_{k+1},T\right),\upsilon_{k+1}\right),\;\ldots \;,H\left(\Phi \left(\eta_{k+1},T\right),\upsilon_{k+1}\right)\right)\in Y^{T} \end{array}$$

The output trajectories $\Gamma (\eta_{k},T)$ and $\Gamma (\eta_{k+1},T)$ up to time T∈N are random variables, making it ineffective to compare their similarities. We define $P_{\eta_{k}}^{T}$ as the distribution law followed by the elements in $\Gamma (\eta_{k},T)$. The distributional observability condition for a stochastic system can be obtained as follows

**Definition 2.**  *At time step k, the UAV transitions from $\eta_{k}$ to $\eta_{k+1}$ under the maneuver $V_{k}$. We state that the target is distributionally observable, and $V_{k}$ is considered an effective maneuver if $P_{\eta_{k}}^{T}\neq P_{\eta_{k+1}}^{T}$.*

Once the UAV maneuver is determined, the distributional observability depends solely on $\epsilon_{k}$ and $\upsilon_{k}$. Distributional observability can be viewed as a generalized extension of deterministic observability. When all noise is ignored, the position of the UAV can be precisely determined, implying that $\Gamma (\eta_{k},T)$ and $\Gamma (\eta_{k+1},T)$ follow the Dirac distribution. In this case, Definition 2 reduces to deterministic observability, as described in Definition 1. Our focus is to determine whether these positions retain distributional observability when process noise and measurement noise are introduced. The relevant notation for the measure space is defined. The Borel σ-algebra is B(S), which is defined in an open set $S\subset R^{n}$, and $M_{1}^{+}\left(S\right)$ indicates probability measures on (S,B(S)). The probability is expressed as (Ω,A,P).

According to (4), UAV measurements are corrupted by *b* and υ. These can be treated as a specialized form of additive noise, denoted as $\hat{\upsilon }$, which follows a Gaussian distribution with mean *b*. The UAV measurement model can thus be expressed as

(22) $$Z_{k}=\arctan\left(\frac{r_{2,k}}{r_{1,k}}\right)+{\hat{\upsilon }}_{k}$$

To represent the influence of $\hat{\upsilon }$, the function K:X×R→Y, $H\left(p_{k},\hat{\upsilon }\right)=K\left(h\left(p_{k}\right),\hat{\upsilon }\right)$ is defined. ${\hat{Z}}_{k}=h\left(P_{k}\right)$ denotes the output before it is corrupted by $\hat{\upsilon }$, and $\hat{\gamma }$ states the output trajectory before it is corrupted by ϵ and $\hat{\upsilon }$. As demonstrated in [43], additive noise does not affect the equivalence of the two distributions. In other words, for any $\mu_{1},\mu_{2}\in M_{1}^{+}\left(Y\right)$ with $\mu_{1}\neq \mu_{2}$, there exists a measurable set A∈B(Y) such that

(23) $$\int_{Y}P\left(K\left(\hat{\gamma },\hat{\upsilon }\right)\in A\right)d\mu_{1}\left(\hat{\gamma }\right)\neq \int_{Y}P\left(K\left(\hat{\gamma },\hat{\upsilon }\right)\in A\right)d\mu_{2}\left(\hat{\gamma }\right)$$

It indicates that the two different distributions, ${\hat{\Gamma }}_{1}$ and ${\hat{\Gamma }}_{2}$, where ${\hat{\Gamma }}_{i}$ represents the output trajectory before it is corrupted by measurement noise, remain distinguishable even after being corrupted by $\hat{\upsilon }$. In subsequent sections, we quantitatively analyze distributional observability via a data-driven approach.

### 3.3. MMD and Quantitative Analysis

In this section, distributional observability is quantified through the MMD algorithm. First, a nonnegative function $k:Y^{T}\times Y^{T}\to R$ is defined as a kernel function, which generates the underlying Hilbert space K defined over $Y^{T}$. It is known as the reproducing kernel Hilbert space (RKHS). We assume that at time step *k*, the elements $\Gamma {\left(\eta_{k},T\right)}_{i}$ in $\Gamma (\eta_{k},T)$ follow the distribution $P_{\eta_{k}}^{T}$, which is mapped to the RKHS via the kernel mean embedding *k* as $p_{k}=E_{X_{i}∼P_{\eta_{k}}^{T}}\left(k\left(\cdot ,X\right)\right)$. Similarly, $P_{\eta_{k+1}}^{T}$ is mapped to $p_{k+1}$. The MMD aims to compute the distance between $P_{\eta_{k}}^{T}$ and $P_{\eta_{k+1}}^{T}$ in the RKHS, which is expressed as follows

(24) $$\mathrm{MMD}\left(P_{\eta_{k}}^{T},P_{\eta_{k+1}}^{T}\right)={∥p_{k}-p_{k+1}∥}_{K}$$

where ${∥\cdot ∥}_{K}$ is the norm in the RKHS space. The Gaussian kernel is used as the kernel function as follows

(25) $$k\left(\Gamma \left(\eta_{k},T\right),\Gamma \left(\eta_{k+1},T\right)\right)=\sum_{i=1}^{T}\sum_{j=1}^{T}\exp\left(-\frac{1}{2\beta^{2}}{\left(\Gamma {\left(\eta_{k},T\right)}_{i}-\Gamma {\left(\eta_{k+1},T\right)}_{j}\right)}^{2}\right)$$

where β>0 is the scalar width of the Gaussian kernel. Assuming that *T* tends to infinity, if $\mathrm{MMD}\left(P_{\eta_{k}}^{T},P_{\eta_{k+1}}^{T}\right)=0$, $P_{\eta_{k}}^{T}$ and $P_{\eta_{k+1}}^{T}$ are identically distributed, then $\eta_{k}$ and $\eta_{k+1}$ exhibit distributional unobservability.

Owing to finite sample constraints (T≪∞), the value of the MMD is computationally approximated through discrete sampling, with the approximated ${\hat{\mathrm{MMD}}}^{2}$ given by

(26) $${\hat{\mathrm{MMD}}}^{2}\left[K,P_{\eta_{k}}^{T},P_{\eta_{k}}^{T}\right]=\frac{1}{T^{2}}k\left(\Gamma_{k},\Gamma_{k}\right)+\frac{1}{T^{2}}k\left(\Gamma_{k+1},\Gamma_{k+1}\right)-\frac{2}{T^{2}}k\left(\Gamma_{k},\Gamma_{k+1}\right)$$

where $\Gamma (\eta_{k},T)$ and $\Gamma (\eta_{k+1},T)$ are abbreviated as $\Gamma_{k}$ and $\Gamma_{k+1}$, respectively. Let the upper bound of the kernel function *k* be B,B∈R with the concentration bound a>0. Inspired by [54], (26) converges in probability to

(27) $$P\left({∥\hat{\mathrm{MMD}}-\mathrm{MMD}∥}_{2}>2\left(\sqrt{\frac{B}{m}}+\sqrt{\frac{B}{m}}\right)+a\right)\leq \exp\left(-\frac{a^{2}m^{2}}{4Bm}\right)$$

By applying the concentration bounds in (27), an acceptable error rate *c* is defined, and the lower bound value κ for MMD is established as follows

(28) $$\hat{\mathrm{MMD}}\left[K,P_{\eta_{k}}^{T},P_{\eta_{k}}^{T}\right]\geq \sqrt{\frac{2B}{m}}\left(1+\sqrt{2\ln c^{-1}}\right)=\kappa$$

According to (28), the definition of distributional observability under finite sampling can be derived as follows

**Definition 3.**  *At time step k, the UAV transitions from $\eta_{k}$ to $\eta_{k+1}$ under the control of the maneuver $V_{k}$. We state that the target is distributionally observable, and $V_{k}$ is considered to be an effective maneuver if ${\hat{\mathrm{MMD}}}^{2}\left[K,P_{\eta_{k}}^{T},P_{\eta_{k}}^{T}\right]\geq \kappa^{2}$.*

**Figure 4:**
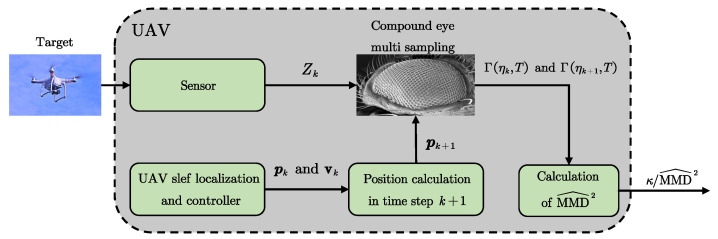
Flowchart of the bio-inspired distributional observability analysis method.

As shown in Definition 3, the ratio $\kappa /\hat{\mathrm{MMD}}$ is considered to be a quantitative metric of distributional observability. The flowchart of the bio-inspired distributional observability analysis method is shown in Figure 4.

Based on the preceding analysis, the performance metric for observability enhancement can be formally derived as

(29) $$\hat{J}={\left(\frac{\kappa }{\hat{\mathrm{MMD}}}\right)}^{2}$$

When $\hat{J}\to 0$, the system exhibits the strongest degree of observability. When $\hat{J}\to 1$, the system exhibits the weakest degree of observability, which is regarded as the distributional unobservability line. The algorithmic pseudocode is presented in Algorithm 1.
**Algorithm 1:** Distributional Observability Quantifying Algorithm
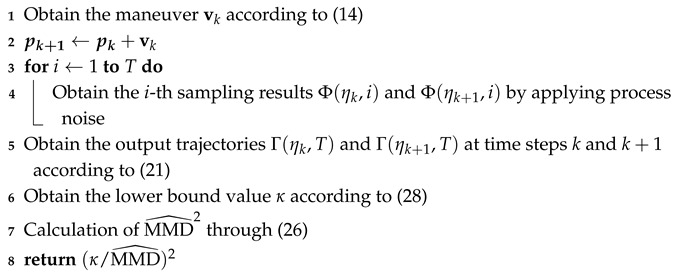


## 4. UAV Trajectory Optimization

This section first introduces the NCMOWOA for solving the nonlinear multi-objective programming problem. The output of the NCMOWOA is then processed to select optimal decision variables. Finally, the flowchart of UAV trajectory optimization is presented.

### 4.1. Optimization Model

Inspired by [44], the region of unobservability is incorporated as a barrier in the control barrier function, and the nonlinear constraint at time step *k* is defined as

(30) $$\begin{array}{c} g\left(u_{k}\right)=\sin\left(2\theta_{k}-2\alpha_{k+1}\right)u_{2,k}+\delta \sin{\left(\alpha_{k+1}-\theta_{k}\right)}^{2}\geq 0\\ \theta_{k}=\theta_{k-1}+u_{2,k}\Delta t \end{array}$$

where $g(u_{k})$ represents the nonlinear constraint of the optimization model at time step *k*, $\alpha_{k+1}$ denotes the UAV’s line-of-sight angle at time step k+1, and δ is a constant in the control barrier function. Subject to the UAV performance constraints, the decision variables $u_{k}$ must comply with specific constraints, which can be expressed as

(31) $$\begin{array}{c} V_{\min}\leq u_{1,k}\leq V_{\max}\\ \omega_{\min}\leq u_{2,k}\leq \omega_{\max} \end{array}$$

Based on the distributional observability, the UAV trajectory optimization model is designed, which is defined as follows

(32) $$\begin{array}{cc} \underset{u_{k}}{\mathrm{Minimize}} & \left\{\begin{array}{c} f_{1,k}\left(u_{k}\right)=\sqrt{{\left(∥r_{k+1}∥-r_{safe}\right)}^{2}}\\ f_{2,k}\left(u_{k}\right)=\hat{J} \end{array}\right.\\ \mathrm{Subject}\;\;\mathrm{to} & \left\{\begin{array}{c} V_{\min}\leq u_{1,k}\leq V_{\max}\\ \omega_{\min}\leq u_{2,k}\leq \omega_{\max}\\ g\left(u_{k}\right)\geq 0 \end{array}\right. \end{array}$$

where $r_{\mathrm{safe}}$ denotes the safe distance between the UAV and the target, and $f_{1,k}$ and $f_{2,k}$ represent the objective functions at time step *k*. The distance function $f_{1,k}$ is used to maintain close UAV–target proximity and the distributional observability metric $f_{2,k}$ is used to improve system state estimation quality. The optimal decision variable $u_{k}$ is obtained by minimizing the objective functions.

Inspired by the bubble-net hunting strategy for humpback whales, the multi-objective nonlinear programming problem in (32) is solved via the NCMOWOA.

### 4.2. Nonlinear Constrained Multi-Objective Whale Optimization Algorithm

In this section, the whale optimization algorithm is first introduced. Then, the fast crowding distance sorting approach and NCESS are described. Finally, the pseudocode of the NCMOWOA is provided.

#### 4.2.1. Whale Optimization Algorithm

Since the global optimum in the search space is unknown a priori, the whale optimization algorithm drives candidate solutions toward the local optimum by simulating unique foraging strategies of humpback whales. The behavior is formulated as follows

(33) $$X_{i}\left(t+1\right)=\left\{\begin{array}{c} \begin{array}{cc} X^{*}\left(t\right)-A\cdot D^{\prime } & \mathrm{if}\;random<0.5\;\mathrm{and}\;\left|A\right|\leq 1 \end{array}\\ \begin{array}{cc} D^{\prime }\cdot e^{ql}\cdot \cos\left(2\pi l\right)+X^{*}\left(t\right) & \mathrm{if}\;random\geq 0.5 \end{array}\\ \begin{array}{cc} X_{rand}\left(t\right)-A\cdot D & \mathrm{if}\;random<0.5\;\mathrm{and}\;\left|A\right|>1 \end{array} \end{array}\right.$$

where *t* is the current iteration, random∈[0,1] and l∈[−1,1] indicate the random number, *q* is a constant for defining the shape of the logarithmic spiral, A≤1 indicates that all the elements in A satisfy $A_{i}\leq 1$ for each component *i*, $X^{*}\left(t\right)$ and $X_{rand}\left(t\right)$ denote the local optimum and a random solution in the current population, respectively, and |·| represents the absolute value. The remaining parameters are defined as follows

(34) $$\begin{array}{c} A=2a\cdot c-a,\;C=2c\\ D=\left|C\cdot X_{rand}\left(t\right)-X_{i}\left(t\right)\right|,\;D^{\prime }=\left|X^{*}\left(t\right)-X_{i}\left(t\right)\right| \end{array}$$

where the elements in a∈[0,2] are linearly decreasing parameters over iterations, and *c* is a random vector and satisfies $c_{i}\in \left[0,1\right]$ for each component *i*.

As formulated in (33), the position vector $X_{i}\left(t\right)$ is updated relative to $X^{*}\left(t\right)$ when random<0.5 and |A|≤1. Through adaptive adjustment of the coefficient vectors A and C, $X_{i}\left(t\right)$ progressively converges toward $X^{*}\left(t\right)$, emulating the prey-encircling behavior of humpback whales. When random≥0.5, a helical equation is formulated between variables $X_{i}\left(t\right)$ and $X^{*}\left(t\right)$ to characterize the spiral movement (exploitation phase) of humpback whales. When random<0.5 and |A|>1, the position vector $X_{i}\left(t\right)$ is deliberately displaced from $X^{*}\left(t\right)$ to model the prey search behavior of humpback whales (exploration phase). Unlike the exploitation phase, the exploration phase adopts a randomly selected candidate solution from the population instead of relying on $X^{*}\left(t\right)$. This mechanism enables the whale optimization algorithm to conduct global exploration, thereby enhancing its ability to approximate the global optimum.

Compared to classical MHAs (genetic algorithm and particle swarm optimization), the whale optimization algorithm achieves a superior exploration–exploitation balance through its spiral bubble-net mechanism, requires fewer hyperparameters, and demonstrates higher robustness in multimodal optimization problems, as validated in structural engineering benchmarks [45,55]. Furthermore, unlike gradient-based methods (MATLAB’s fmincon (version R2023b)), the whale optimization algorithm’s derivative-free nature avoids local optima traps and accommodates non-differentiable observability metrics, making it ideal for UAV trajectory optimization.

#### 4.2.2. Fast Crowding Distance Sorting Approach

For the single-objective optimization scenario, the local optimum $X_{t}^{*}$ in the population can be directly selected based on the objective function. Multi-objective scenarios present challenges for selecting the local optimum. The set of all solutions is the feasible solution set Ω. In the context of the optimization problem aimed at minimizing the objective function, $X_{1}\in \Omega$ is said to dominate $X_{2}\in \Omega$, which can be formalized as

(35) $$X_{1}≺X_{2}\;\;\mathrm{if}\;\;\exists i\in \left\{1,2\right\}:\;f_{i,k}\left(X_{1}\right)\;<f_{i,k}\left(X_{2}\right)$$

A solution $X_{1}\in \Omega$ is the Pareto optimal solution if and only if there does not exist another solution X∈Ω such that

(36) $$\forall i\in \left\{1,2\right\}:f_{i,k}\left(X\right)\leq f_{i,k}\left(X_{1}\right)\;\mathrm{and}\;\exists j\in \left\{1,2\right\}:f_{j,k}\left(X\right)<f_{j,k}\left(X_{1}\right)$$

**Figure 5:**
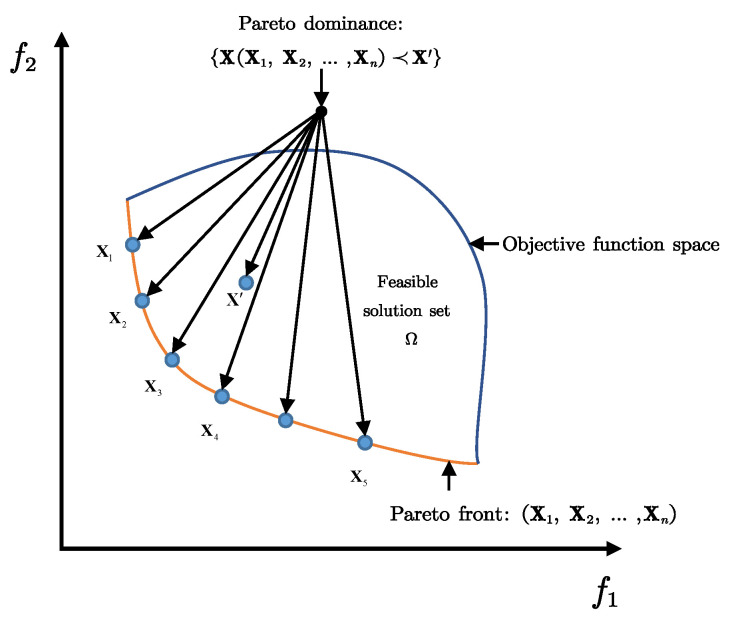
Definitions in the search space of a two-objective optimization problem.

The Pareto front is the set of all Pareto optimal solutions in Ω. The relevant concepts of multi-objective domination and the associated terminology are illustrated in Figure 5.

**Figure 6:**
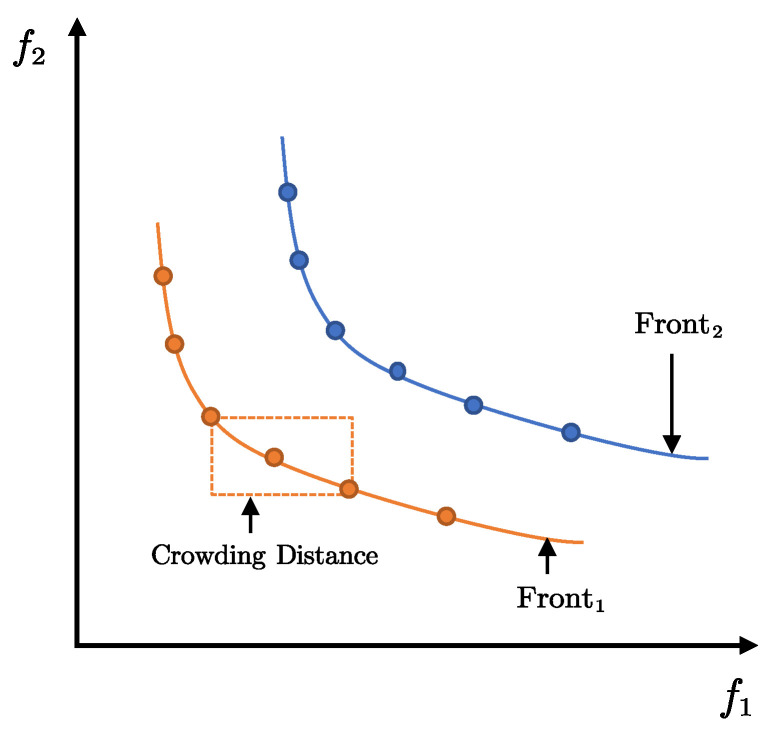
Visualization of crowding distances.

The multiple objectives we consider are inherently conflicting. No single solution can simultaneously optimize all objectives, making Pareto analysis crucial for understanding these trade-offs. The Pareto front provides mission planners with a spectrum of optimal solutions representing different objective weightings. This allows the selection of the most appropriate decision variables. According to the Pareto front, the local optimum $X^{*}\left(t\right)$ is calculated as follows

(37) $$X^{*}\left(t\right)=\frac{X^{best1}\left(t\right)+X^{best2}\left(t\right)+X^{best3}\left(t\right)}{3}$$

where $X^{best1}\left(t\right)$, $X^{best2}\left(t\right)$, and $X^{best3}\left(t\right)$ are the top three optimal solutions in the Pareto front. Inspired by [47], the fast crowding distance sorting approach is utilized to classify the population into multiple ranks, where the solutions within the same rank do not dominate each other. The Pareto front corresponds to $rank_{1}$. The relationship between the ranks is as follows: $rank_{i}$ dominates all the solutions in $rank_{j}$ (i<j). As shown in Figure 6, the Pareto front contains multiple solutions. To measure the quality of individual solutions on the same frontier, the crowding distance (CD) [56] is assigned to each solution to rank them.

The calculation of the CD requires sorting the solutions in ascending order. The solution bounds are set to (−∞,∞), representing unbounded constraints in the optimization space. The CD for intermediate solutions is computed as the absolute normalized difference between the function values of the neighboring solutions. Maintaining a certain CD is beneficial for preserving population diversity, and the crowding distance of each solution can be expressed as

(38) $$dis\left[i\right]=\sum_{k=1}^{dim}\frac{f_{k}\left[i+1\right]-f_{k}\left[i-1\right]}{f_{k}^{\max}-f_{k}^{\min}}$$

where dim is the dimension of the object function, and $f_{k}\left[i+1\right]$ and $f_{k}\left[i-1\right]$ represent the values of two adjacent solutions in the *k*-th dimension. CD sorting does not rely on parameter settings or optimization model downscaling to maintain the diversity of solutions in the population. $X^{best1}\left(t\right)$, $X^{best2}\left(t\right)$, and $X^{best3}\left(t\right)$ are selected based on CD sorting, which ensures population diversity during the optimization process.

The local optimum $X^{*}\left(t\right)$ identified through CD sorting does not satisfy the optimal decision variable condition that maximizes the contribution to the system. It can only be used to represent the diversity of the population. Inspired by [57,58], the optimal decision variable is selected through the technique for order preference by similarity to ideal solution (TOPSIS) and the criteria importance through intercriteria correlation (CRITIC). First, the Pareto front is obtained via the fast nondominated sorting method. Next, the objective weights are dynamically determined at each iteration through the CRITIC method. The CRITIC method determines weights based on the similarities and differences among the objective functions. Finally, the values of the objective functions are synthesized and evaluated via the TOPSIS method.

#### 4.2.3. Nonlinear Constrained Elitist Selection Strategy

**Figure 7:**
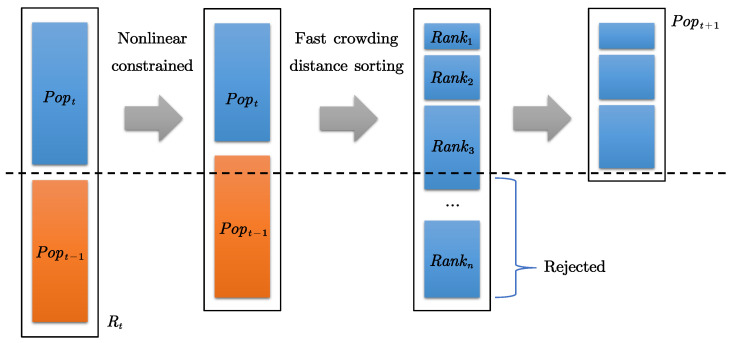
Flowchart of NCESS.

The nonlinear constraints pose significant challenges in multi-objective optimization problems. The multi-objective whale optimization algorithm does not inherently account for these nonlinear constraints, which can result in solutions being updated beyond the feasible boundaries. To address this limitation, we introduce the elitist selection strategy and incorporate the nonlinear constraint. The process flow is illustrated in Figure 7.

First, the parent population $Pop_{t-1}$ and the offspring population $Pop_{t}$ are combined into a new population $R_{t}$ with a size of 2N. Then, the nonlinear constraint is applied to $Pop_{t}$. Subsequently, the constrained population undergoes fast crowding distance sorting, which decomposes it into multiple fronts and ranks them based on crowding distance to obtain Rank. Finally, *N* solutions are selected in order of merit to form the population $Pop_{t+1}$. The pseudocode of the NCMOWOA is shown in Algorithm 2.

In line 3, $T_{p}$ is the maximum iteration time. Lines 1 to 2 define the various categories of variables required by the algorithm and complete the population initialization process. Specifically, in line 2, $Archive_{F}$ is initialized as an infinite matrix to ensure it does not influence the results of the first iteration. The algorithm updates the population via the whale optimization algorithm from lines 6 to 13.

The algorithm skips the nonlinear constraint during the first iteration to prevent the $Archive_{X}$ and $Archive_{F}$ from being deleted by nonlinear constraints. $Combined_{X}$ and $Combined_{F}$ are used to record the mixing results of the offspring and the parent populations. The next-generation solutions, $Archive_{X}$ and $Archive_{F}$, are subsequently obtained through the NCESS. After completing the iteration in line 20, the optimal decision variable $u_{k}$ is selected in $Archive_{X}$ through the TOPSIS-CRITIC method, as the output of the NCMOWOA.
**Algorithm 2:** NCMOWOA
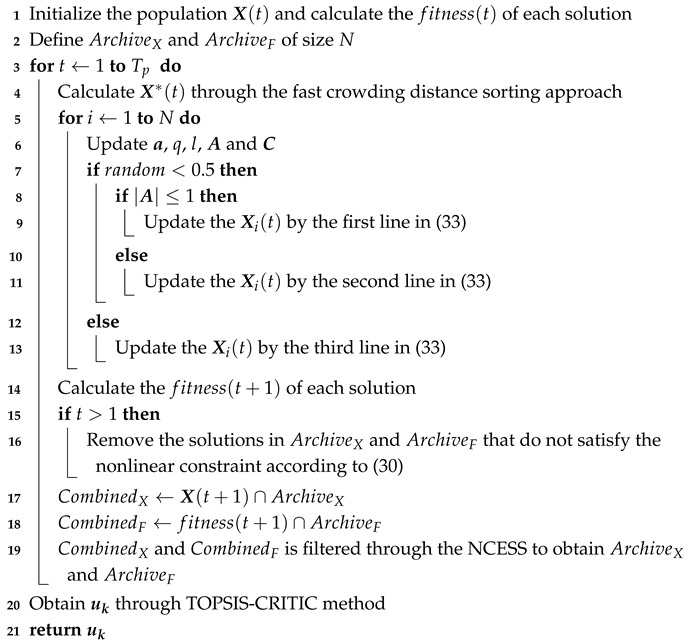


### 4.3. The Flowchart of UAV Trajectory Optimization

**Figure 8:**
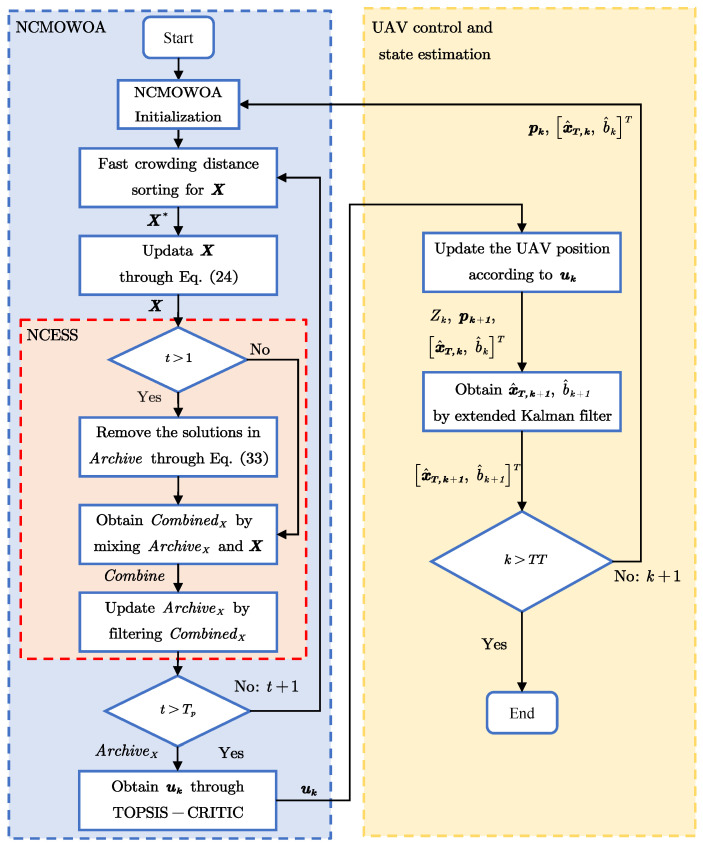
Flowchart of UAV trajectory optimization.

The flowchart of the UAV trajectory optimization method is shown in Figure 8, where TT is the maximum simulation time. The method is composed of three modules: NCMOWOA, NCESS, and UAV control and state estimation. First, $X^{*}$ is obtained via fast crowding distance sorting and X is updated by 33 in the NCMOWOA module. Second, the NCESS module, which is a critical component of the NCMOWOA, screens the updated X and obtains the $Archive_{X}$. When the number of iterations *t* exceeds the maximum iteration time $T_{p}$, the optimal decision variable is filtered by the TOPSIS-CRITIC method. The UAV control and state estimation module subsequently updates the UAV position $p_{k}$ based on the Pareto optimal solution and obtains the new estimation $p_{k+1},{\left[{\hat{x}}_{T,k+1},{\hat{b}}_{k+1}\right]}^{T}$ via the extended Kalman filter. Finally, the UAV position $p_{k+1}$ and the state estimation ${\left[{\hat{x}}_{T,k+1},{\hat{b}}_{k+1}\right]}^{T}$ are input to the NCMOWOA module to begin the next round. Otherwise, the simulation is terminated.

## 5. Results

In this section, we validate the effectiveness of the proposed UAV trajectory optimization method. Firstly, the effect of noise on distributional observability was verified. Secondly, the localization accuracy and convergence of the proposed algorithm were evaluated. Thirdly, we analyzed the differences in target localization and sensor bias estimation between the proposed method and the approach in [26]. Finally, the performance of the NCMOWOA was evaluated via comparisons with the MOPSOA [46], NSGA-II [47], MOEDOA [48], and NSGA-III [49]. All MMD calculations employ the Gaussian kernel function, and the parameters are presented in Table 1.

### 5.1. Influence of Noise on Distributional Observability

**Figure 9:**
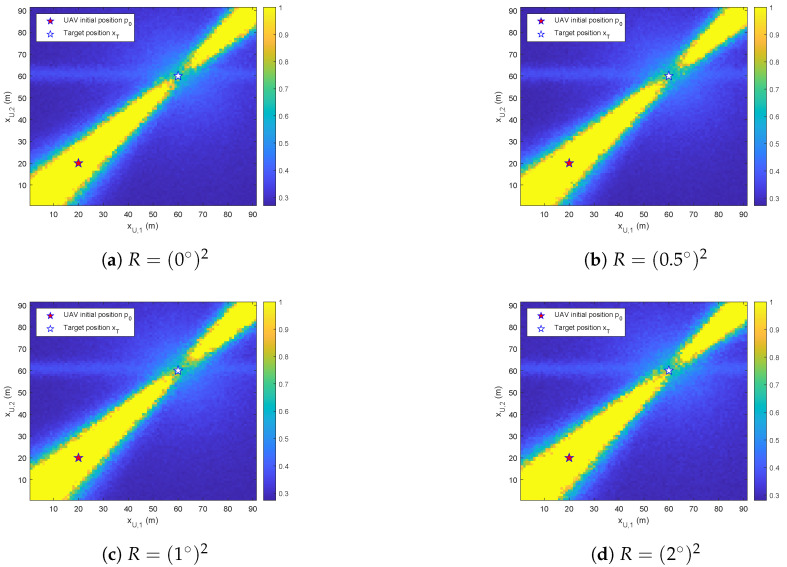
The $\kappa /\hat{\mathrm{MMD}}$ on the $x_{U}$-grid under the influence of measurement noise with variances *R*.

We select the UAV’s initial position as $p_{0}=\left(20\;m,20\;m\right)$ and the target’s position as $x_{T}=\left(60\;m,60\;m\right)$. Then, a 90m×90m guide with a resolution of 1m is constructed. The distributional observability of the UAV was evaluated under the influence of process noise with covariance $Q=diag(3^{2},3^{2})$. We calculate the metric $\kappa /\hat{\mathrm{MMD}}$ for the output trajectories of the UAV from $p_{0}$ to each grid point, as depicted in Figure 9.

Figure 9 illustrates the distributional observability under the influence of measurement noise with varying variances *R*. The region of distributional unobservability, defined by $\kappa /\hat{\mathrm{MMD}}>1$, is labeled as 1 for presentational clarity. We observe that the system exhibits regions of distributional unobservability (error rate *a*) along the $p_{0}-x_{T}$ axis. The distributional observability is shown to improve proportionally with the displacement from this axis.

Since $\hat{\mathrm{MMD}}$ is derived from limited data, Figure 9a–d can be considered approximately equivalent. This demonstrates that the distributional observability of the system is not affected by measurement noise. To maintain consistent parameters, a simulation was conducted to evaluate the influence of process noise when measurement noise was excluded.

**Figure 10:**
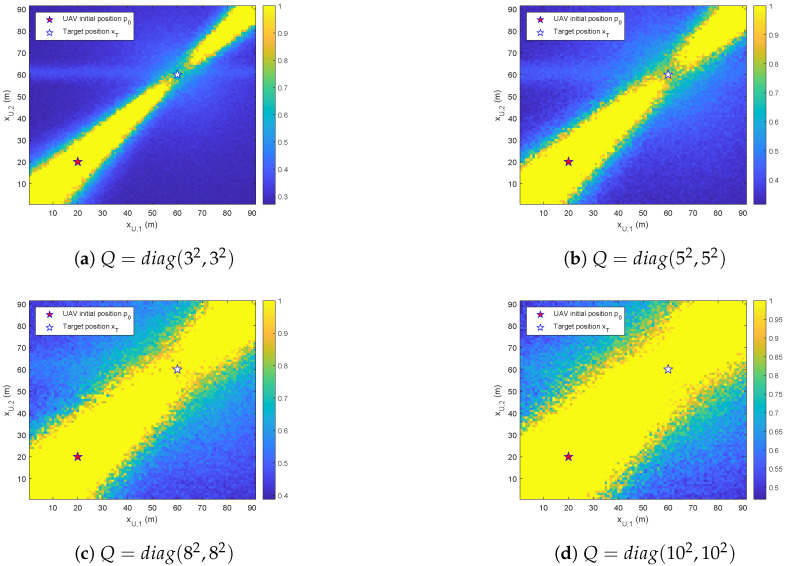
The $\kappa /\hat{\mathrm{MMD}}$ on the $x_{U}$-grid under the influence of process noise with variances *Q*.

Figure 10 illustrates the distributional observability under the influence of process noise with varying variances *Q*. A pronounced expansion of the unobservable region is observed with elevated process noise covariance. Consequently, a larger maneuver is required for the UAV to escape these regions and ensure sufficient observability for maintaining localization accuracy.

### 5.2. Trajectory Optimization Results

The target is assumed to be stationary, and the state transition matrix $W_{k+1,k}$ is defined as an identity matrix. The simulation parameters are presented in Table 2.

The state estimation ${\left[{\hat{x}}_{T,1},{\hat{x}}_{T,2},\hat{b}\right]}^{T}$ is obtained via an extended Kalman filter. The initial state estimation and the error covariance COV are initialized as

(39) $${\left[{\hat{x}}_{T,1},{\hat{x}}_{T,2},\hat{b}\right]}^{T}=\left[\begin{array}{c} 280\\ 280\\ 10^{\circ } \end{array}\right]\;\;COV=\left[\begin{array}{ccc} {\left(15\right)}^{2} & 0 & 0\\ 0 & {\left(15\right)}^{2} & 0\\ 0 & 0 & {\left(0.1^{\circ }\right)}^{2} \end{array}\right]$$

**Figure 11:**
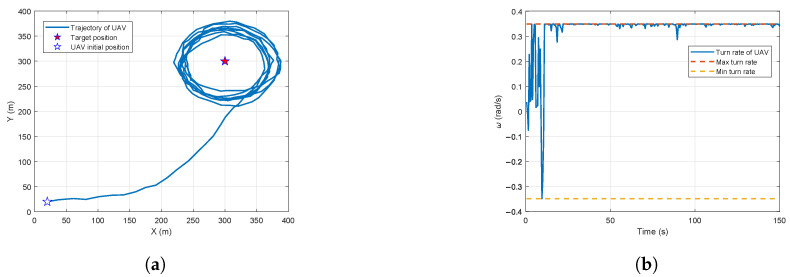
Trajectory optimization results for the UAV flight trajectory and turn rate: (**a**) UAV flight trajectory. (**b**) Turn rate.

The measurements are calibrated in real time through the estimation $\hat{b}$, effectively mitigating its systematic impact. In this closed-loop mode, the corrected measurement can be expressed as

(40) $$Z_{k,close}=Z_{k}-{\hat{b}}_{k-1}$$

where ${\hat{b}}_{k-1}$ represents the estimated measurement bias vector at the previous time step. The results of the UAV flight trajectory and turn rate are shown in Figure 11.

**Figure 12:**
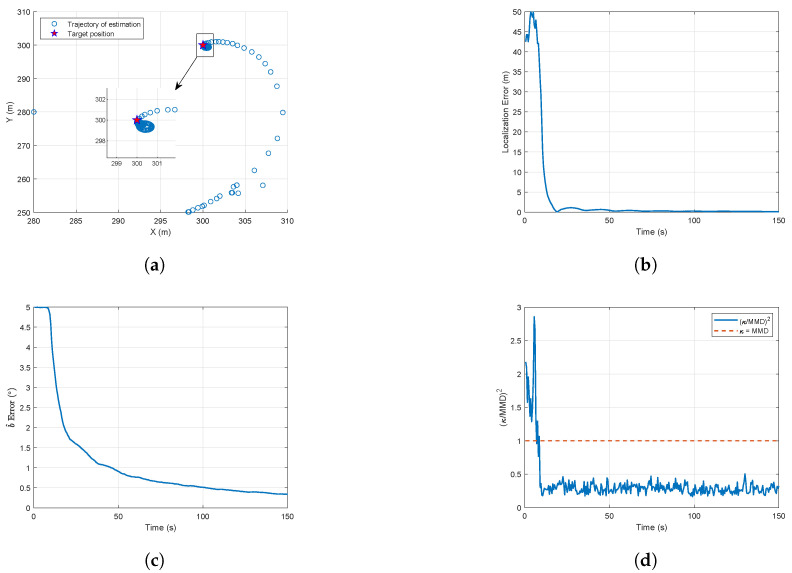
Trajectory optimization results for state estimation and distributional observability: (**a**) Target position estimation trajectory. (**b**) Localization error. (**c**) Bearing bias estimation error. (**d**) Distributional observability.

In Figure 11a, the UAV first tends toward the target to reduce the relative distance, and then converges to an approximately circular orbit, where process noise slightly varies from a circular path. Figure 11b shows the variation in the UAV turn rate, indicating that the UAV turn rate converges to the maximum turn rate and remains relatively stable. To verify the variation in state estimation over time, the results for state estimation and distributional observability are shown in Figure 12.

In Figure 12a, the estimated position of the target approaches the true position finally. Figure 12d demonstrates the gradual convergence of the distributional observability quantitative metric, ${\left(\kappa /\hat{\mathrm{MMD}}\right)}^{2}$, below the distributional unobservability line $\left({\left(\kappa /\hat{\mathrm{MMD}}\right)}^{2}=1\right)$ during the trajectory optimization process. While the MMD is an empirical value computed from finite data and subject to small fluctuations, these fluctuations remain below the unobservability threshold. As shown in Figure 12b,c, as ${\left(\kappa /\mathrm{MMD}\right)}^{2}$ converges, the target localization error and estimation error of *b* gradually decrease.

### 5.3. Comparison of $\hat{J}$ and *J*

**Figure 13:**
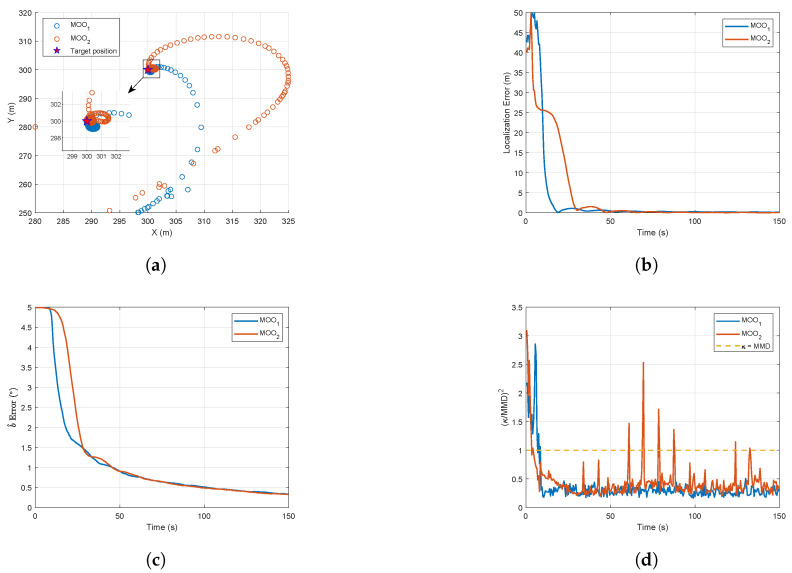
Comparison results between ${\mathrm{MOO}}_{1}$ and ${\mathrm{MOO}}_{2}$: (**a**) Target position estimation trajectories. (**b**) Localization error. (**c**) Bearing bias estimation error. (**d**) Distributional observability.

In this section, we conducted a comparative performance analysis between the proposed method and the approach in [26], specifically examining their behavior under process noise. The corresponding optimization model is formulated as follows

(41) $${\mathrm{MOO}}_{1}=\left[\begin{array}{c} \hat{J}\\ {∥r-r_{\mathrm{safe}}∥}_{2} \end{array}\right],\;{\mathrm{MOO}}_{2}=\left[\begin{array}{c} J\\ {∥r-r_{\mathrm{safe}}∥}_{2} \end{array}\right]$$

where ${\mathrm{MOO}}_{1}$ denotes the optimization model proposed in this work, and ${\mathrm{MOO}}_{2}$ corresponds to the model derived from (16). For a fair comparison, the nonlinear constraint and optimization methods in the NCMOWOA are incorporated into ${\mathrm{MOO}}_{2}$. Its relevant parameters are shown in Table 2 and the comparison results are illustrated in Figure 13.

Figure 13a demonstrates that ${\mathrm{MOO}}_{1}$ achieves a tighter estimation trajectory radius than ${\mathrm{MOO}}_{2}$. Figure 13b depicts the convergence characteristics of the localization error, revealing that the localization error for ${\mathrm{MOO}}_{1}$ converges significantly faster than that of ${\mathrm{MOO}}_{2}$. Figure 13c indicates that, in the pre-optimization phase, the $\hat{b}$ of ${\mathrm{MOO}}_{1}$ is more precise than that of ${\mathrm{MOO}}_{2}$, potentially contributing to its accelerated convergence behavior. Figure 13d presents the distributional observability of ${\mathrm{MOO}}_{1}$ and ${\mathrm{MOO}}_{2}$. The performance of ${\mathrm{MOO}}_{1}$ and ${\mathrm{MOO}}_{2}$ was evaluated through integral of time-weighted absolute error (ITAE) computation for localization error, bearing bias estimation, and distributional observability, with results shown in Table 3.

The quantitative comparison reveals ${\mathrm{MOO}}_{1}$’s consistent superiority over ${\mathrm{MOO}}_{2}$ across all ITAE metrics. Compared with ${\mathrm{MOO}}_{2}$, which is based on the separation angle, the optimization model proposed in this paper significantly improves UAV target localization performance under the influence of process noise.

### 5.4. Comparison with Other Metaheuristic Algorithms

In this section, the NCMOWOA was compared with other MHAs in terms of efficiency. All MHAs are configured with the same parameters, as shown in Table 2. The parameters unique to the MHAs in this study are presented in Table 4.

**Figure 14:**
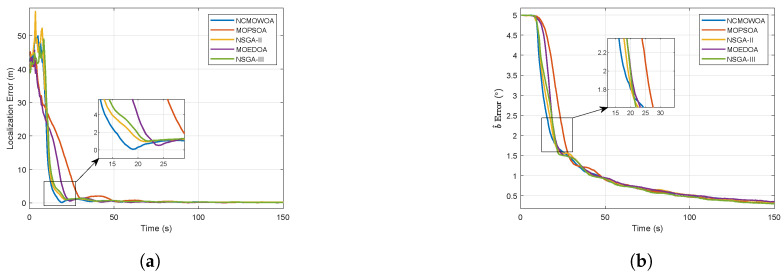
Simulation results with other MHAs: (**a**) Localization error. (**b**) Bearing bias estimation error.

**Figure 15:**
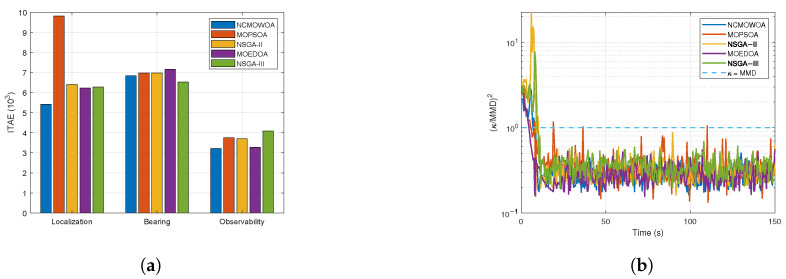
(**a**) ITAE. (**b**) Distributional observability.

The simulation results are presented in Figure 14. As shown in Figure 14a, the NCMOWOA converges faster than the other MHAs in the initial phase, and its final localization error is close to 0. Figure 14b presents the bearing bias estimation error, demonstrating that the NCMOWOA achieves reduced estimation errors during the initial phase compared to other MHAs. To better compare the performance of MHAs, the ITAE and distributional observability were calculated, as shown in Figure 15.

Figure 15a demonstrates that the NCMOWOA attains the minimal distributional observability ITAE among all MHAs. The comparative performance analysis in Figure 15b reveals the NCMOWOA’s convergence characteristics. To comprehensively assess algorithm performance, GD and IGD were employed to evaluate the convergence characteristics and comprehensive performance of MHAs, which can be expressed as

(42) $$\begin{array}{cc} \mathrm{GD}=\frac{\sqrt{\sum_{i=1}^{\left|Pareto\right|}d_{i}^{2}}}{\left|Pareto\right|} & \mathrm{IGD}=\frac{\sqrt{\sum_{j=1}^{\left|Pareto^{*}\right|}{\hat{d}}_{j}^{2}}}{\left|Pareto^{*}\right|} \end{array}$$

where Pareto denotes the approximate Pareto front obtained by the MHA, $Pareto^{*}$ represents the true Pareto front, |Pareto| indicates the cardinality of the obtained solution set, and $d_{i}=\min_{y^{*}\in Pareto^{*}}{∥y_{i}-y^{*}∥}_{2}$ is the minimum Euclidean distance between the $i^{th}$ solution in Pareto and the true Pareto front, while ${\hat{d}}_{j}=\min_{y\in Pareto}{∥y_{j}^{*}-y∥}_{2}$ denotes the minimum Euclidean distance from the $j^{th}$ reference solution in $Pareto^{*}$ to the approximate front. The lower the GD value, the better the convergence performance, while a reduced IGD value indicates the superior comprehensive performance of the MHA. To ensure comparability, the Pareto front is normalized within [0,1] for each objective.

The performance of MHAs was assessed through the ITAE, GD, and IGD. The corresponding results are presented in Table 5.

In Table 5, the three performance metrics localization error, $\hat{b}$ error, and distributional observability are evaluated via ITAE. The NCMOWOA significantly outperforms other MHAs in both localization error and distributional observability, as evidenced by its consistently lower ITAE values. Regarding the approximate Pareto front, the NCMOWOA achieves superior convergence and diversity, as demonstrated by its significantly smaller GD and IGD values compared to other MHAs. These results provide theoretical validation for the algorithm’s outstanding performance.

## 6. Conclusions

This paper investigates the trajectory optimization problem for UAV target localization with biased bearing-only measurement. Firstly, based on the geometric condition, a distributional observability analysis method is proposed for stochastic systems. Then, a UAV trajectory optimization framework is proposed by constructing a quantitative metric for observability enhancement. Within this framework, the UAV maneuvers are determined through multi-objective optimization, and the target localization and sensor bias are estimated simultaneously via an extended Kalman filter. Finally, numerical simulations and comparative analyses are conducted to validate the analytical results. The comparative analysis demonstrates the superior performance of the proposed optimization model $MOO_{1}$ in both target localization accuracy and sensor bias estimation. Quantitative evaluations through the ITAE, GD, and IGD metrics reveal that the NCMOWOA significantly outperforms competing MHAs in terms of solution diversity and convergence characteristics.

## Figures and Tables

**Table 1:** MMD-related parameters.

Parameters	Value
Scalar width β	1
Error rate *a*	5%
Upper bound *B*	1
MMD data size *m*	100
Measurement expansion	8

**Table 2:** Parameters for trajectory optimization.

Parameters	Value
Sampling time Δt	0.5 s
Maximum simulation time TT	150 s
Process noise covariance matrix *Q*	$diag(5^{2},5^{2})$
Safe distance $r_{safe}$	50 m
Target position $x_{T}$	(300 m, 300 m)
Initial position of UAV $p_{0}$	(20 m, 20 m)
Bearing bias *b*	$5^{\circ }$
Range of velocity	[10 m/s, 40 m/s]
Range of turn rate	[$-20^{\circ }/s$, $20^{\circ }/s$]
Measurement noise variance *R*	${\left(0.5^{\circ }\right)}^{2}$
Maximum population size *N*	50
Maximum iteration time $T_{p}$	30
*q* in (33)	1
δ in (30)	0.12

**Table 3:** ITAE for localization error, bearing bias estimation error, and distributional observability.

Mode	ITAE of ${\mathrm{MOO}}_{1}$/$10^{3}$	ITAE of ${\mathrm{MOO}}_{2}$/$10^{3}$
Localization error	5.42	8.80
Bearing bias error	6.83	7.20
Distributional observability	3.22	4.61

**Table 4:** Parameters of other MHAs.

Parameters	Value
Inertia weight in MOPSOA	0.45
Acceleration constants in MOPSOA	(1.2, 1.2)
Crossover rate in NSGA-II	0.9
Mutation rate in NSGA-II and NSGA-III	0.1

**Table 5:** Performance comparison between MHAs.

Mode	NCMOWOA	MOPSOA	NSGA-II	MOEDOA	NSGA-III
Localization error$/10^{3}$	5.42	9.82	6.40	6.23	6.28
$\hat{b}$ error$/10^{3}$	6.83	6.98	6.99	7.17	6.53
Distributional observability$/10^{3}$	3.22	3.75	3.70	3.28	4.09
GD/$10^{-2}$	3.99	5.87	4.89	6.53	5.07
IGD/$10^{-2}$	5.27	7.55	7.52	8.62	6.90

## Data Availability

The data are contained within the article.

## Abbreviations

The following abbreviations are used in this manuscript:

UAV	Unmanned aerial vehicle
MMD	Maximum mean discrepancy
NCESS	Nonlinear constrained elitist selection strategy
MHA	Metaheuristic algorithm
NCMOWOA	Nonlinear constrained multi-objective whale optimization algorithm
MOPSOA	Multi-objective particle swarm optimization algorithm
NSGA-II	Nondominated sorting genetic algorithm II
MOEDOA	Multi-objective exponential distribution optimization algorithm
NSGA-III	Nondominated sorting genetic algorithm III
RKHS	Reproducing kernel Hilbert space
CD	Crowding distance
TOPSIS	Technique for order preference by similarity to ideal solution
CRITIC	Criteria importance through intercriteria correlation
ITAE	Integral of time-weighted absolute error
GD	Generational distance
IGD	Inverted generational distance
